# The analysis of organic diseases in elderly patients diagnosed with bipolar disorders- A retrospective study

**DOI:** 10.1192/j.eurpsy.2023.1480

**Published:** 2023-07-19

**Authors:** O. Vasiliu

**Affiliations:** Psychiatry Department, Dr. Carol Davila University Emergency Central Military Hospital, Bucharest, Romania

## Abstract

**Introduction:**

Both organic and psychiatric comorbidities are frequently detected in bipolar disorders (BD) and this phenomenon has a significant impact on case management in these patients. The most reported general medical conditions in patients with BD are migraine, thyroid illness, obesity, diabetes mellitus, and cardiovascular disease. The screening for comorbidities detection in BD patients is important from multiple perspectives, including the need to construct an adequate therapeutic plan, the need to increase treatment adherence, and to improve these patients’ quality of life.

**Objectives:**

To assess the prevalence of comorbid organic diseases in a clinical sample of BD patients.

**Methods:**

A chart- and register-based analysis of BD patients (i.e., type I and II BD) evaluated in our department during a period of three years (January 2019- January 2022) was conducted in order to detect the prevalence rate of organic diseases. All patients aged over 65 years were evaluated either for acute mood episodes or presented to their treating psychiatrists for follow-up visits.

**Results:**

A number of 87 patients were included in this analysis, 45 male and 42 female, with a mean age of 70.5 years. The most frequently detected organic comorbidities were metabolic disorders (obesity, dyslipidemia, diabetes mellitus- 36.8%), cardiovascular (arterial hypertension, arrhythmias, myocardial ischemia, deep/superficial venous thrombosis- 26.4%), digestive system-related (hepatic, gastric, intestinal diseases- 21.8%), neurologic (i.e., stroke, migraine, Parkinson’s disease) (18.3%), endocrine (mainly thyroid diseases) (17.2%), and other (19.5%). A relatively high rate of organic diseases (38%) were newly diagnosed (in the last 3 months), suggesting there is a certain need to screen for organic pathology in this population, but most of the BD patients presented a long history of investigated and treated comorbid conditions.

**Conclusions:**

Organic diseases are very common in elderly BD patients. Even where no history of somatic conditions exists, comprehensive investigations are granted in order to detect such health problems with important prognostic impact.

**Disclosure of Interest:**

None Declared

